# Transcriptome and Metabolome Analysis Provide New Insights into the Process of Tuberization of *Sechium edule* Roots

**DOI:** 10.3390/ijms23126390

**Published:** 2022-06-07

**Authors:** Lihong Su, Shaobo Cheng, Yuhang Liu, Yongdong Xie, Zhongqun He, Mingyue Jia, Xiaoting Zhou, Ruijie Zhang, Chunyan Li

**Affiliations:** 1 College of Horticulture, Sichuan Agricultural University, Chengdu 611130, China; sulihong@stu.sicau.edu.cn (L.S.); 17835417978@163.com (S.C.); liuyuhang_edu@stu.sicau.edu.cn (Y.L.); manulll3my@163.com (M.J.); zhouxiaoting6412@hotmail.com (X.Z.); dflydjk@163.com (R.Z.); sicaulcy521@163.com (C.L.); 2 Institute for Processing and Storage of Agricultural Products, Chengdu Academy of Agricultural and Forest Sciences, Chengdu 611130, China; xieyd1135@163.com

**Keywords:** gene expression, *Sechium edule*, metabolomics, tuber yield, tuberization, transcriptomics

## Abstract

Chayote *(Sechium edule)* produces edible tubers with high starch content after 1 year of growth but the mechanism of chayote tuberization remains unknown. ‘Tuershao’, a chayote cultivar lacking edible fruits but showing higher tuber yield than traditional chayote cultivars, was used to study tuber formation through integrative analysis of the metabolome and transcriptome profiles at three tuber-growth stages. Starch biosynthesis- and galactose metabolism-related genes and metabolites were significantly upregulated during tuber bulking, whereas genes encoding sugars will eventually be exported transporter (SWEET) and sugar transporter (SUT) were highly expressed during tuber formation. Auxin precursor (indole-3-acetamide) and ethylene precursor, 1-aminocyclopropane-1-carboxylic acid, were upregulated, suggesting that both hormones play pivotal roles in tuber development and maturation. Our data revealed a similar tuber-formation signaling pathway in chayote as in potatoes, including complexes BEL1/KNOX and SP6A/14-3-3/FDL. Down-regulation of the BEL1/KNOX complex and upregulation of 14-3-3 protein implied that these two complexes might have distinct functions in tuber formation. Finally, gene expression and microscopic analysis indicated active cell division during the initial stages of tuber formation. Altogether, the integration of transcriptome and metabolome analyses unraveled an overall molecular network of chayote tuberization that might facilitate its utilization.

## 1. Introduction

Chayote (*Sechium edule*) originated in Central America and Mexico and is taxonomically classified within Cucurbitaceae [1,2]. It is cultivated in tropical and subtropical regions and is now a commercial crop worldwide [3]. Similar to other Cucurbitaceous species, *S. edule* is a herbaceous climbing plant with tendrils and unisexual flowers. The edible fruits are consumed as vegetables and are also known as chayote, chuchu (Brazil), christophene, sayote (Philippines), and mirliton [3,4]. There are 10 chayote species reported to date, and the fruit flavor varies among cultivars from bland to sweetish or starchy [5]. Additionally, the fruit reportedly has high nutritional value owing to its high contents in vitamin C, folic acid, certain essential amino acids, minerals, phenolics, flavonoids, and carotenoids [4,5,6,7]; all of which, make it one of the most consumed imported products in the United States, Canada, and the European Union, after tomato, avocado, and coffee [3].

In addition to fresh-eating, Ke et al. [8] optimized a well ultrasound-assisted extraction condition to extract chayote pectin which results in a low degree of esterification, high molar mass, and suitable foaming capacity. This provides beneficial use of chayote pectin as a gelling, stabilizing, thickening, and emulsifying agent in the food industry. The tender leaves and stems of cultivated *S. edule* which contain an amount of protein, pectin, and lipids, are also frequently consumed daily [9]. Clinical and epidemiological studies have revealed the role of chayote fruit composition in the prevalence of chronic diseases [10,11]. Chayote shoots composition flavonoids were shown to decrease serum lipids and cholesterol content, preventing atherosclerosis [12] and fatty liver and modulating the hepatic lipid metabolism [13].

In addition to the fruit, the edible tuber, which is produced after chayote plants have grown for at least one year, is a morphological feature that makes chayote rather special among Cucurbitaceous species. The starch content of chayote tubers is 73 g/100 g (on a dry weight (DW) basis), which is similar to that of potato tubers (70 g/100 g DW) and they taste much like potatoes after cooking [5,14]. Chayote tubers are white and contain small amounts of slowly digestible and resistant starch, and large amounts of rapidly digestible starch, which makes it highly appreciated throughout Latin American countries [5]. In addition, chayote tuber starch can be used as a thickening agent in food dispersions due to its high viscosity [15].

With the advanced sequencing technology, a large amount of data resources and research methods have been applied to study the chayote plant. Fu et al. (2021) [16] used Nanopore third-generation sequencing combined with Hi–C data to assemble a draft chayote genome and estimated the genome size as 606.42 Mb. Meanwhile, complete chloroplast genome sequence of the *S. edule* had been reported [17]. These research established genome bases for further chayote study. 

To the best of our knowledge, primarily fruits of the reported chayote cultivars are consumed but there are no edible tuber-producing commercial chayote cultivars. A previous study revealed genes and metabolites involved in fruit texture, pigment, flavor, flavonoids, antioxidants, and plant hormones during chayote fruit development by transcriptional and metabolic analysis [16]. However, there are few published reports on chayote tuber formation. Therefore, the objective of this study was to introduce a chayote cultivar that produces edible tubers and to unravel the theoretical basis of chayote tuberization.

## 2. Results

### 2.1. Morphological Characteristics of S. edule cv. ‘Tuershao’

**Figure 1:**
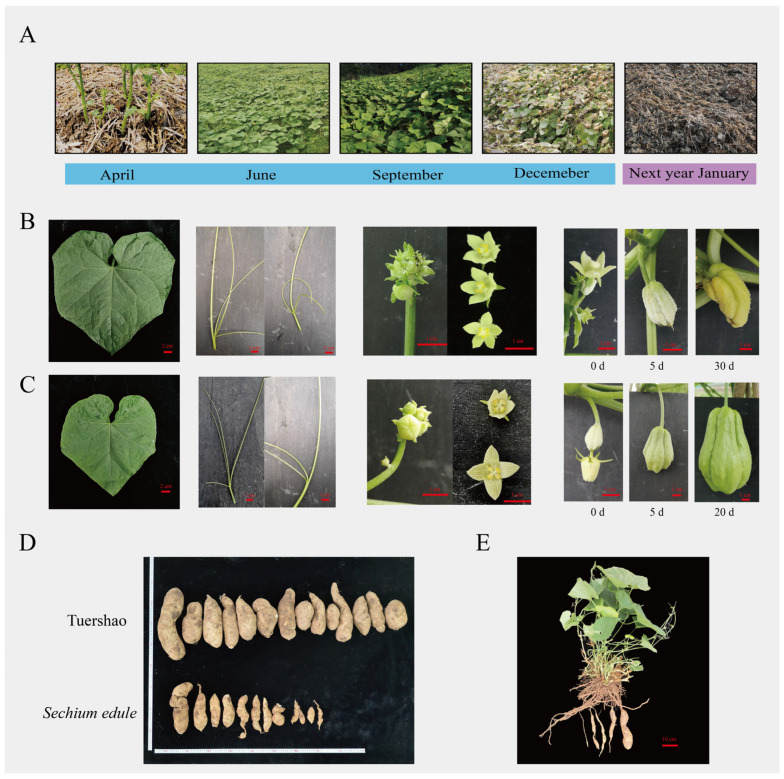
Morphological characteristics of ‘Tuershao’ and *S. edule*. ‘Tuershao’ plant growth in one growing season (**A**). Morphological characteristics of ‘Tuershao’ (**B**) and *S. edule* (**C**). Tuber yield after one year of growth of ‘Tuershao’ and four-year-old *S. edule* plants (**D**). ‘Tuershao’ plant (**E**).

‘Tuershao’ chayote plants were cultivated in Ya’an City, Sichuan Province (30°06′ N, 102°75′ E), which sprouted in April and completely senesced in January of the following year (Figure 1A). The morphological features of ‘Tuershao’ (Figure 1B,C) are similar to those of the traditional cultivated *S. edule*. The leaves of ‘Tuershao’ and *S. edule* are ovate-cordate to suborbicular and contain three to four separate tendrils. The flowers of ‘Tuershao’ and *S. edule* are unisexual (Figure 1B,C) and contain both male and female reproductive organs on the same plant. The male flowers of ‘Tuershao’ and *S. edule* are clustered and contain four to five stamens and petals.

‘Tuershao’ and *S. edule* showed major differences in flowering time, female development, and tuber yield. In Sichuan province, *S. edule* plants showed two flowering periods in one year, first, from May to July and then from September to November. ‘Tuershao’ plants only had one flowering period in a year in Sichuan province, from September to November. The ovary of ‘Tuershao’ cannot grow into a fruit (Figure 1B,C). Meanwhile, its tuber yield after one year of growth and that of four-year-old *S. edule* plants averaged 10.77 and 2.33 kg per plant, respectively. Therefore, we concluded that ‘Tuershao’ is a specific chayote cultivar with a high yield of edible tubers.

### 2.2. Starch Content and Soil Physical Properties during Tuber Formation

**Figure 2:**
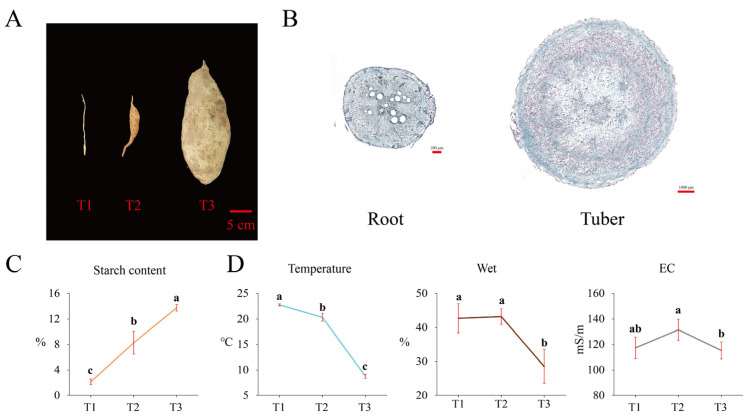
Starch accumulation and soil physical properties at three different stages of tuber growth. Photographs of tubers at stages T1, T2, and T3 (**A**). Microscopic view of a root and a tuber cross-section (**B**). Starch content (**C**) and soil physical properties at stages T1, T2, and T3 (**D**).

Tubers at three different growth stages (T1, T2, and T3) are shown in Figure 2A. The microscopy of samples collected at stages T1 and T2 (Figure 2B) revealed high starch accumulation during tuberization. We determined starch content at three stages (Figure 2C), which significantly increased during tuber formation and reached 13.76% (on fresh weight, FW, basis) at stage T3. Additionally, we measured the physical properties of the soil (Figure 2D) at the same three stages. Soil temperature significantly decreased with time, and soil moisture at T3 was significantly lower than that at either T1 or T2. Thus, we speculate that low temperature might contribute to the starch accumulation and tuber formation.

### 2.3. Transcriptome Characterization during Tuberization

**Figure 3:**
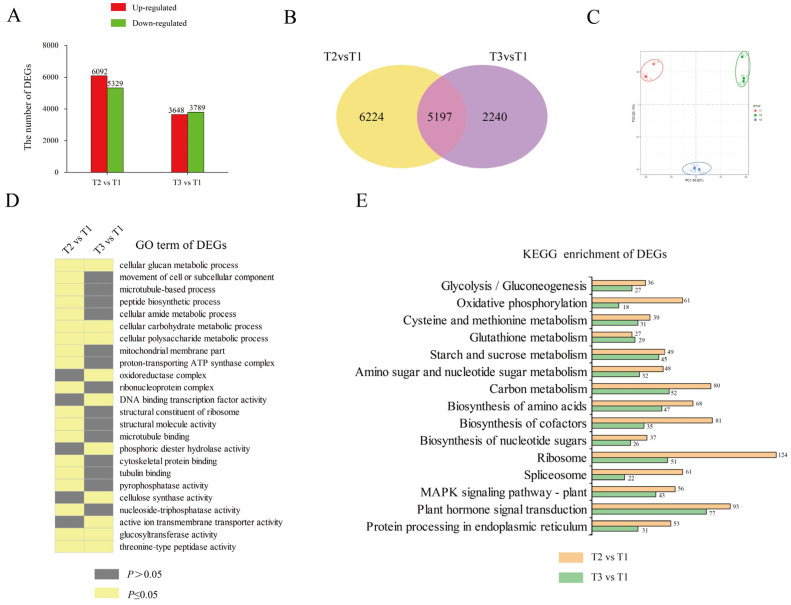
RNA-seq analysis of tubers at three different growth stages. DEGs in different comparisons (**A**); red bars represent upregulated genes and green bars represent downregulated genes. Venn diagram of DEGs in different comparisons (**B**). PCA analysis of samples at different growth stages (**C**). GO enrichment analysis of DEGs (**D**). Number of DEGs in each KEGG pathway (**E**).

We performed RNA-seq to profile the dynamic changes in gene expression during tuberization of ‘Tuershao’. Transcriptomes at T2 and T3 were compared with those at T1 to identify differentially expressed genes (DEGs). A total of 11421 and 7437 DEGs were identified in T2 vs. T1 and in T3 vs. T1, respectively, with 6092 and 3648 upregulated DEGs, and 5329 and 3789 downregulated DEGs, respectively (Figure 3A). There were many more DEGs in T2 vs. T1 than in T3 vs. T1, indicating strong gene network variation at tuber initiation.

To elucidate the gene-network response to tuberization, GO and KEGG enrichment analyses were performed to determine their biological functions (Figure 3C,D). In the GO term, DEGs were enriched in ‘cellular carbohydrate metabolic process’, ‘cellular polysaccharide metabolic process’, ‘glucosyltransferase activity’, and ‘cellular glucan metabolic process’. Meanwhile, in the KEGG enrichment pathway, most DEGs were enriched in ‘plant hormone signal transduction’, ‘starch and sucrose metabolism’, ‘biosynthesis of amino acids’, and ‘carbon metabolism’. These results revealed that, during tuberization, DEGs were mainly involved in glycometabolism and metabolite biosynthesis.

### 2.4. Characterization of the Metabolome of Tuberization

**Figure 4:**
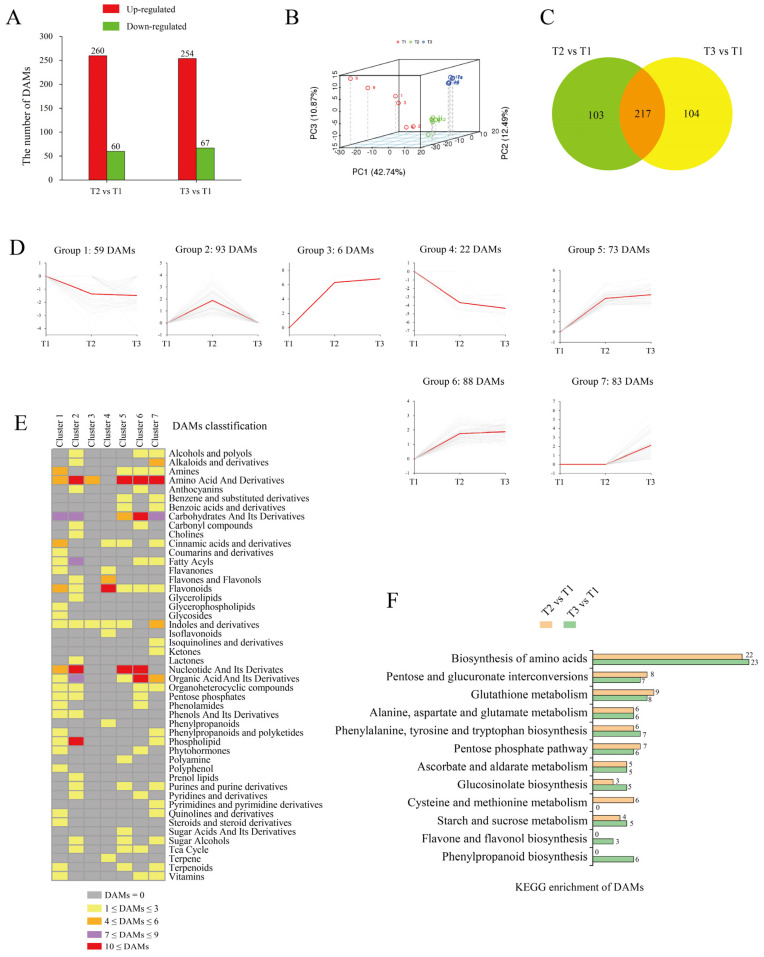
Metabolome data from tubers in different comparisons. Number of DAMs in different comparisons (**A**); red bars represent upregulated metabolites and green bars represent downregulated metabolites. PCA of samples at different growth stages (**B**). Venn diagram of DAMs (**C**). K-mean cluster analysis of DAMs (**D**); in each group, gray lines represent the pattern of metabolite accumulation and red lines represent the accumulation trend of all metabolites. DAMs classification of each group (**E**). Number of DAMs in each KEGG pathway (**F**).

To compare the metabolite contents in ‘Tuershao’ tubers at different stages of growth, we analyzed tuber samples by HPLC-MS/MS at three sampling time points (T1, T2, and T3). Metabolites at T2 and T3 were analyzed for comparison with those at T1, and we set |Log2 (foldchange)| ≥ 1, *p*-value < 0.05, and VIP (variable importance in project) ≥ 1 as thresholds for differentially accumulated metabolites (DAMs). A total of 320 and 321 DAMs were identified between T2 vs. T1 and T3 vs. T1, with 260 and 254 upregulated DAMs and 60 and 67 downregulated DAMs, respectively (Figure 4A). The PCA (Figure 4B), showed that tuber samples from the three different growth stages were separated by PC1 (42%), PC2 (12%), and PC3 (10%). The Venn diagram showed 217 DAMs at stages T2 and T3, compared to T1 (Figure 4C). 

K-means cluster analysis was applied to obtain an overall picture of metabolite changes at the three tuber growth stages under study (Figure 4D). DAMs at T2 and T3 clustered into seven groups. According to the accumulated metabolite trend, these seven groups were further subdivided into four forms: form I (decrease–decrease, including groups 1 and 4), form II (increase–increase, including groups 3, 5, and 6), form III (increase–decrease, group 2), and form IV (constant–increase, group 7). We then classified the metabolites in each group (Figure 4E). First, 81 DAMs were grouped into form I, which were mainly classified into flavonoids, amines, cinnamic acids, and derivatives. Then, a total of 167 DAMs were grouped into form II, which were mainly classified into amino acids and derivatives, carbohydrates and their derivatives, nucleotides and their derivatives, and organic acids and their derivatives. Finally, 93 DAMs were grouped into form III, which were mainly classified into fatty acyls, organic acids and their derivatives, and phospholipids. These 260 metabolites, which were grouped into forms II and III, accumulated at the early stage (T2) of tuberization. Thereafter, 83 DAMs were grouped into form Ⅳ, which were mainly classified into alkaloids and derivatives, amino acids and derivatives, indoles and derivatives, and carbohydrates and their derivatives. These 83 metabolites accumulated at the terminal stage (T3) of tuberization. 

Metabolome KEGG enrichment analysis (Figure 4F) revealed that DAMs were involved in the biosynthesis of amino acids, pentose and glucuronate interconversions, glutathione metabolism, pentose phosphate pathway, ascorbate and aldarate metabolism, and starch and sucrose metabolism. Thus, most DAMs are associated with carbohydrate and amino acid metabolism. 

### 2.5. Plant-Hormone Pathways Response to Tuberization

**Figure 5:**
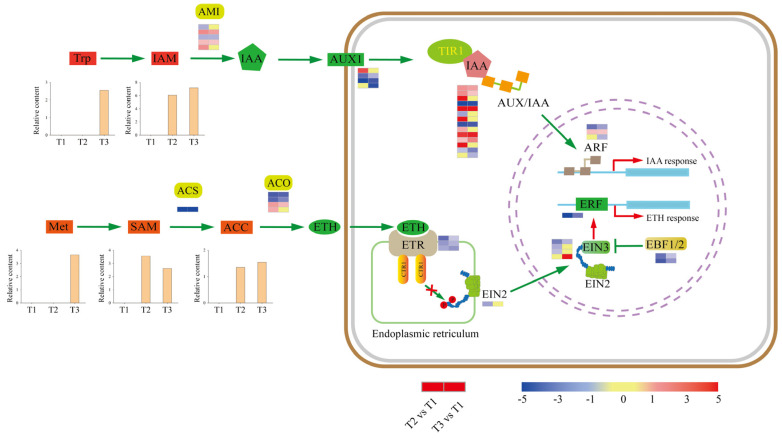
DEGs and DAMs in plant hormone biosynthesis and signal transduction. The expression heat maps from left to right are T2 vs. T1 and T3 vs. T1.

KEGG analyses revealed that DEGs were enriched in plant hormone signal transduction. Thus, we screened DEGs and DAMs involved in plant hormone pathways (Figure 5). The results showed that auxin (IAA) precursor [indole-3-acetamide (IAM)] and ethylene (ETH) precursor [1-aminocyclopropane-1-carboxylic acid (ACC)] were upregulated, suggesting that IAA and ETH might play vital roles in tuberization. 

The indole acetic acid metabolism (IAM) pathway is presumably widely distributed in plants, although exactly how IAM is produced from the amino acid tryptophan (Trp) remains unclear. The conversion of IAM to IAA is catalyzed by AMIDASE 1 (AMI1). Five DEGs encoding AMI1 were identified, with three upregulated and two downregulated genes at the T2 stage, and two upregulated and one downregulated gene at the T3 stage. In IAA signal transduction, auxin influx carriers (AUX1) contribute to the movement of IAA into the cytoplasm. Then, IAA promotes the association of auxin/indole acetic acid repressor (Aux/IAA) proteins and the TRANSPORT INHIBITOR1 (TIR1) ubiquitin ligase complex, resulting in the repression of AUXIN RESPONSE FACTOR (ARFs) to activate or repress gene expression (Figure 5). Four DEGs encoding AUX1 were identified during tuberization, among which one was upregulated and two were downregulated at the T2 stage, while three were downregulated at the T3 stage. Additionally, fourteen genes encoding Aux/IAA proteins were identified, of which nine were upregulated and four were downregulated at stage T2, while five were upregulated and four were downregulated at stage T3. Three DEGs encoding ARFs were identified, one was upregulated and one was downregulated at the T2 stage, one was upregulated and two were downregulated at stage T3. These results suggest a tuberization-promoting role for IAA.

ETH is formed from the amino acid methionine (Met) in three subsequent steps (Figure 5). First, S-adenosyl methionine (SAM) synthetase converts Met to SAM. Then, 1-aminocyclopropane-1-carboxylic acid synthase (ACS) transforms SAM to 1-aminocyclopropane-1-carboxylic acid (ACC). DEGs encoding ACS synthetase was downregulated at stages T2 and T3 during tuber formation. Finally, ACC is oxidized by ACC oxidase (ACO) to produce ETH. Four DEGs encoding ACO were identified, with two upregulated and two downregulated at stage T2, and one upregulated and two downregulated at stage T3. Overall, metabolome data showed that ETH precursors SAM and ACC were upregulated at stages T2 and T3. 

In ETH signal-transduction (Figure 5), ETHYLENE RESPONSE1 (ETR1), CONSTITUTIVE TRIPLE RESPONSE 1 (CTR1), ETHYLENE INSENSITIVE 2 (EIN2), ETHYLENE INSENSITIVE 3 (EIN3), and ethylene response factors (ERF1) are the major proteins involved in this process. Three DEGs encoding ETR1 were downregulated at stages T2 and T3. Four DEGs encoding EIN3 were identified, three of which were downregulated at the T2 stage, one upregulated and one downregulated at stage T3. DEGs encoding ERFs were downregulated during tuber formation. EIN3 BINDING F-BOX PROTEIN 1 and 2 (EBF1 and 2) are known as regulators of EIN3. In our study, DEGs encoding EBF1/2 were downregulated at T2 and T3 stages.

### 2.6. Sucrose Metabolism during Tuberization

**Figure 6:**
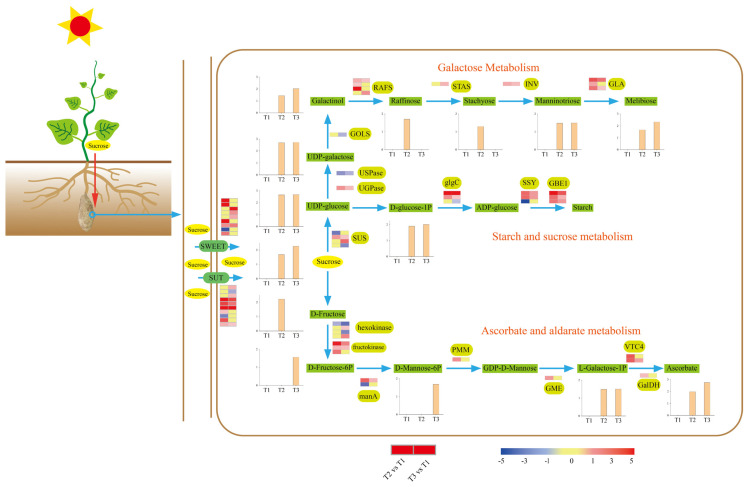
Schematic diagram of sucrose metabolism in ‘Tuershao’ tubers. Expression heat maps from left to right are T2 vs. T1 and T3 vs. T1.

Starch detection and microscopy results revealed high starch accumulation during tuberization. Meanwhile, KEGG enrichment analysis showed that abundant DEGs and DAMs were involved in carbohydrate metabolism, starch metabolism, and sucrose metabolism. Hence, we scanned our transcriptome and metabolome data and revealed the carbohydrate metabolic process during tuberization (Figure 6).

**Figure 7:**
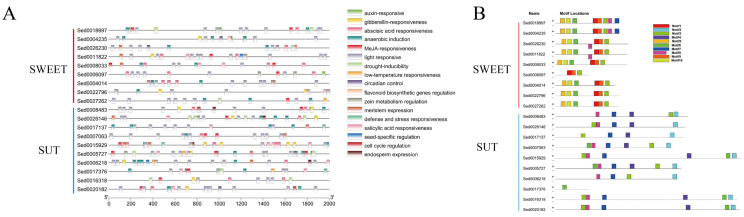
Cis-acting elements analysis (**A**) and protein conserved motif (**B**) of SWEET and SUT genes.

Sucrose is the main photosynthetic product transported from source to sink tissues in most plants. Thus, sucrose was considered the original metabolic substrate in the tuber. The SUGARS WILL EVENTUALLY BE EXPORTED TRANSPORTER (SWEET) family and sugar transporter (SUT) can transport sucrose across the plasma membrane. Nine SWEET and ten SUT DEGs were identified, most of which were upregulated during tuberization (Figure 6). The promoter region of SWEET and SUT DEGs was analyzed to study the potential regulatory network (Figure 7). The results show that all the SWEET and SUT DEGs contain the light response element. Three SWEET and three SUT DEGs contain the low-temperature responsiveness element. Two SWEET and two SUT DEGs contain the auxin-responsive element. Six SWEET and eight SUT DEGs contain abscisic acid responsiveness elements. Protein conserved motif analysis shows that both SWEET and SUT genes exhibited a conserved motif 3 except *Sed0017376*. These results indicated that short photoperiod, low temperature, and plant hormones might play an important role in ‘Tuershao’ sugar transportation during tuber formation.

Tuber sucrose content was upregulated at stages T2 and T3. Sucrose synthase (SUS) catalyzes the reversible cleavage of sucrose into UDP-glucose and fructose. Four DEGs encoding SUS were identified, with one upregulated and one downregulated at stage T2, and two upregulated and one downregulated at stage T3. Through the starch and sucrose metabolism pathway, UDP-glucose is converted to starch by glucose-1-phosphate adenylyltransferase (glgC), starch synthase (SSY), and 1,4-alpha-glucan branching enzyme (GBE1). Most DEGs involved in this pathway were upregulated at stages T2 and T3. These results provide a clear picture of the gene network responsible for the modulation of the high starch content in ‘Tuershao’ chayote tubers (Figure 6).

UDP-galactose is a substrate of the galactose metabolism pathway, which is supplied by UDP-glucose catalyzed by UTP-glucose-1-phosphate uridylyltransferase (UGPase) and UDP-sugar pyrophosphorylase (USPase), and can be transformed into manninotriose and melibiose through the galactose metabolism pathway. DEGs encoding UGPase were upregulated, and DEGs encoding USPase were downregulated at T2 and T3. Using myo-inositol and UDP-galactose as substrates, GALACTINOL SYNTHASE (GOLS) catalyzes the production of galactinol, whereas RAFFINOSE SYNTHASE (RAFS) uses sucrose and galactinol to synthesize raffinose. One DEG encoding GOLS was identified and downregulated at T3. DEGs encoding RAFS were all upregulated at T2 and T3. Through the catalysis of stachyose synthetase (STAS), beta-fructofuranosidase (INV), and alpha-galactosidase (GLA), raffinose was converted to stachyose, manninotriose, and melibiose. DEGs encoding these three enzymes were all upregulated during tuberization (Figure 6).

In addition to carbohydrate metabolism, sucrose was converted to ascorbate, which was upregulated during tuberization via ascorbate and aldarate metabolism (Figure 6). Fourteen DEGs were identified to participate in ascorbate biosynthesis, most of which (78.6%) were upregulated at T2 and T3 (Figure 6). These results revealed a transformed network from sucrose to other nutritional metabolites.

### 2.7. Identification of Transcription Factors during Tuber Formation

**Figure 8:**
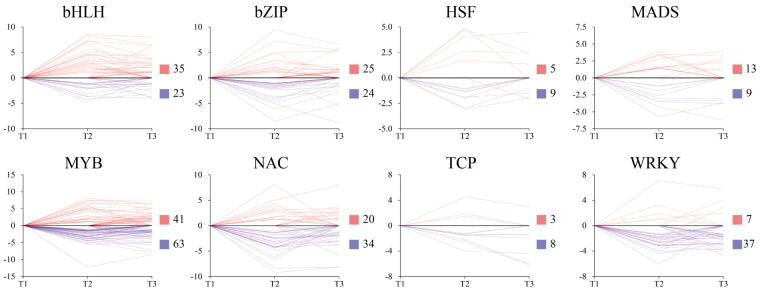
Expression patterns of different transcription factors during tuberization in ‘Tuershao’. Y-axis denotes log_2_(Foldchange). Red and blue squares represent up and downregulated genes, respectively.

Transcription factors (TFs) participate in multiple plant growth and developmental processes. We scanned RNA-Seq data and identified 356 differentially expressing TFs from 8 TF families during tuber formation (Figure 8). Among these 8 TF families, MYB (60.6%), WRKY (84.1%), NAC (62.9%), and TCP (72.7%) exhibited downregulation during tuber formation. In contrast, more than half of the TFs from the bHLH (60.3%) and MADS-box (59.1%) families were upregulated.

### 2.8. Signal Network Regulating Tuberization

**Figure 9:**
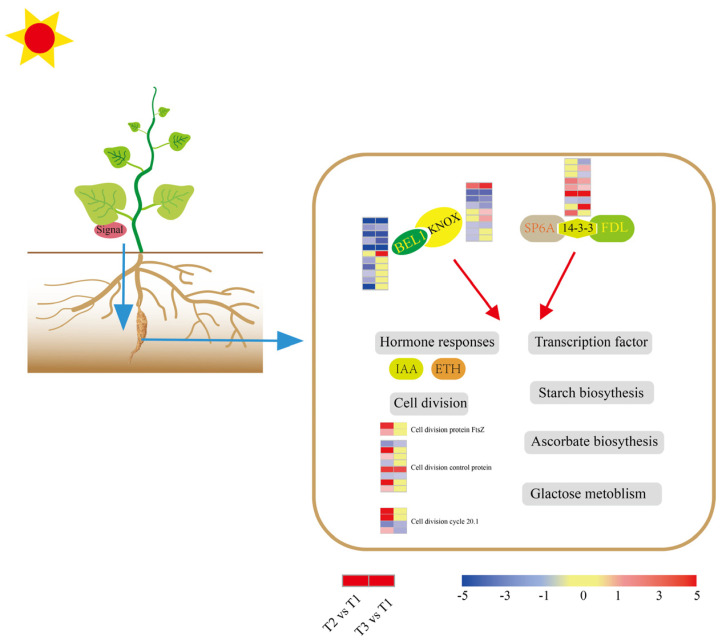
Model of the tuber signaling network. The expression heat maps from left to right are T2 vs. T1 and T3 vs. T1.

To identify the signal that mediates ‘Tuershao’ tuberization, we referenced the protein regulators of tuber formation in potatoes. Eleven BELLRINGER-1-like (BEL1) TFs were identified, with ten downregulated at T2, five downregulated and one upregulated at T3 (Figure 9). In plants, BEL1-like proteins function in a tandem complex with KNOTTED1-LIKE HOMEOBOX (KNOX) proteins. Nine KNOX TFs were identified, with six downregulated and one upregulated at T2, four downregulated, and three upregulated at T3. The 14-3-3 potato protein blocks flowering and promotes tuberization. Nine DEGs encoding 14-3-3 proteins were identified, with four upregulated and one downregulated at T2, and five upregulated and three downregulated at T3. Fourteen DEGs related to cell division were identified, including cell division protein FtsZ, cell division cycle 20.1, and cell division control protein (CDCP). Among them, DEGs encoding cell division protein and cell division control protein were mainly (71.4%) upregulated during the initiation of tuberization.

### 2.9. Validation of DEGs by qRT-PCR

**Figure 10:**
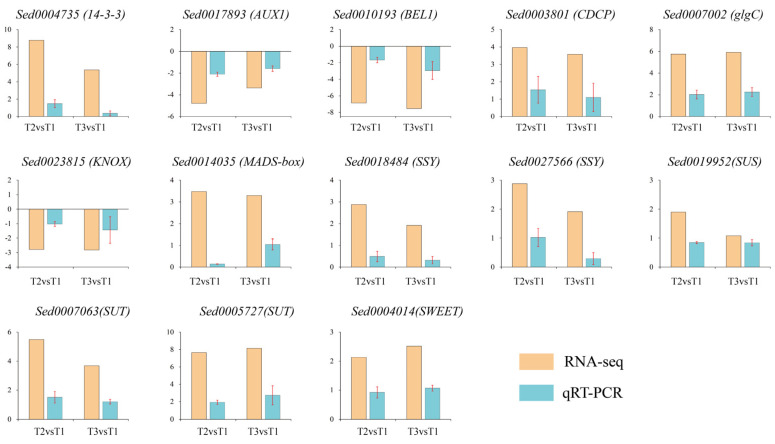
qRT-PCR verification of DEGs, where yellow bars represent RNA-seq data (log_2_FoldChange) and blue bars represent qRT-PCR data (log_10_2^−ΔΔCT^).

To confirm the RNA-seq results, thirteen DEGs that have different roles in ‘Tuershao’ tuber formation were selected. A significant correlation was observed between the qRT-PCR and RNA-seq results (Figure 10), indicating that reliable RNA-seq data were obtained from the samples.

## 3. Discussion

Root and tuber crops including potato, yam, carrot, cassava, sweet potato, turnip, and ginger, among many others, play an important role in human nutrition, providing essential carbohydrates, proteins, and vitamins [18]. After growing for at least one year, plants of *S. edule* produce edible tubers; thus, the species might be developed as a tuber crop due to the high starch content that can accumulate in its tubers [5,14]. However, to date, the known chayote cultivars are primarily grown for their fruit, whereas there are no edible tuber-producing commercial chayote cultivars. Here, we report a high tuber-yield chayote cultivar, named ‘Tuershao’, characterized by a 13.7% starch content (FW) in mature tubers. Transcriptome and metabolome analyses were conducted at three different stages of tuber growth to establish the overall network of chayote tuberization.

In potatoes, the FLOWERING LOCUS T (FT) ortholog, designated StSP6A, is a prominent mobile tuber signal [19] that forms a protein complex with StFDL1 (FLOWERING LOCUS D) via the 14-3-3 protein, which appears to function as a tuber-activation complex (TAC) [20]. Further, overexpression of StSP6A induced early tuberization in a 14-3-3-dependent manner, whereas suppression of StFDL1 reportedly delayed tuberization [20]. In our study, most DEGs encoding 14-3-3 were upregulated at stages T2 and T3 of tuber growth, indicating that a similar TAC might operate in chayote tubers (Figure 9). Additionally, TF BEL5 also serves as a signal in the potato tuberization pathway [21]. Thus, the movement of BEL5 from the leaves to distal organs has been correlated with increased tuber yields [22]. In potatoes, StBEL5 interacts with StKNOX (KNOTTED1-LIKE HOMEOBOX) proteins in a tandem complex to regulate tuberization [21]. Consistently, DEGs encoding BEL1-like and KNOX proteins were identified in our study, most of which were downregulated at T2 and T3. These results indicated that a similar signal network controlling tuberization might be at play in chayote (Figure 9). Therefore, we speculate that the BEL1-like/KNOX complex promotes chayote tuber induction at the initial stage, while the SP6A/FDL/14-3-3 complex plays a crucial role in tuber swelling.

Storage root-bulking generally involves an increase in size and weight. Enhancement in storage root size occurs due to the increase in cell number and cell size, while the storage root weight increases due to the accumulation of starch [23,24]. During ‘Tuershao’ tuber formation, several DEGs involved in cell division were identified and most of them (71.4%) were upregulated at the initial bulking stage. In the starch biosynthesis pathway, starch is synthesized from sucrose and the enzymes involved in this process were wildly studied, including sucrose synthase (SUS), glucose-1-phosphate adenylyltransferase (glgC), starch synthase (SSY), and 1,4-alpha-glucan branching enzyme (GBE1) [25,26,27]. Most of these genes were highly expressed during sweet potato bulking [28]. A similar phenomenon was observed in our study. In galactose metabolism, UDP-glucose-4-epimerase (UALE), GALACTINOL SYNTHASE (GOLS), RAFFINOSE SYNTHASE (RAFS), stachyose synthetase (STAS), beta-fructofuranosidase (INC), and alpha-galactosidase (GLA), catalyze the conversion of sucrose into raffinose, stachyose, manninotriose, and melibiose [29,30,31]. Most DEGs and DAMs in this process were upregulated, despite a significant decrease in ambient temperature. Indeed, a previous study reported that raffinose, stachyose, manninotriose, and melibiose accumulate rapidly at low temperatures [32,33]. These results underline the importance of low temperature during ‘Tuershao’ tuberization to obtain tubers with high nutritional value (Figure 6).

Sucrose is the main photosynthetic product transferred from source to sink organs such as roots, flowers, fruits, and seeds, to enable their growth and development [27,34]. In this process, SWEET and SUT proteins are major sugar transporters across cellular membranes [35]. Thus, for example, mutants of both maize (ZmSWEET4) and its rice ortholog (OsSWEET4) demonstrated that SWEET4 functions as a hexose transporter enhancing sugar import into the endosperm [36]. Consistently, OsSWEET11 is strongly expressed in the ovular vascular trace, nucellar epidermis, and cross cells, whereas knockout of OsSWEET11 significantly reduces sucrose concentration in the embryo sacs, leading to defective grain filling in rice [37]. Meanwhile, ossweet14:ossweet11 and ossweet15:ossweet11 double knockout mutants show more severe phenotypes than ossweet11 single-knockout mutants, indicating a complementary function between SWEET proteins [38,39]. During ‘Tuershao’ tuberization, nine DEGs encoding SWEET proteins were identified, most of them (88.9%), upregulated. Several studies have revealed that SUT1, SUT2, and SUT4 are expressed in tubers and play important roles in tuber development [40,41]. In our study, most DEGs encoding SUT genes were upregulated (Figure 6). These results imply that high expression of SWEET and SUT1 facilitate sucrose transfer from leaves to tubers and the subsequent starch accumulation.

The process of tuber formation involves multiple molecular and cellular changes that are reportedly associated with the levels and/or actions of various phytohormones [42,43]. Thus, for example, IAA has been extensively studied in relation to this process, and IAA levels reportedly increase during the early tuberization stage, presumably acting to stimulate cell division and trigger the storage sink development process [44,45]. In general, plants have several pathways to produce IAA from Trp [46]. Here, our metabolome analysis revealed that IAM was upregulated at stages T2 and T3, compared to T1, which indicated that IAA biosynthesis might proceed through the IAM pathway in ‘Tuershao’ during tuber initiation. Using loss-of-function mutants, Lakehal et al. [47] showed that three Aux/IAA proteins, encoded by IAA6, IAA9, and IAA17, respectively, interacted with positive adventitious root (AR) formation regulators, ARF6 and/or ARF8, thereby inhibiting AR initiation. In our study, AUX/IAA was mainly upregulated, which might result in weak AR initiation during tuberization.

In addition to IAM, the ETH precursor ACC was also upregulated during ‘Tuershao’ tuberization. Li et al. [48] reported that ETH and ACC have distinct functions and suggested that ACC is a signaling molecule in *Marchantia*. Furthermore, Vanderstraeten et al. [49] demonstrated that ACC functions as a growth regulator during early vegetative development and that it plays a role as an ethylene precursor. Thus, ACC might play a special role in ‘Tuershao’ tuberization, independently of ETH; a hypothesis that warrants further exploration. In ETH signal-transduction, ETHYLENE INSENSITIVE 3 (EIN3) binds to the promoter of ethylene response factors (ERF1) to modulate ethylene-responsive genes. In turn, EIN3 BINDING F-BOX proteins 1 and 2 (EBF1 and 2) target EIN3 for proteolysis [50]. During ‘Tuershao’ tuberization, DEGs encoding EINs and ERF were expressed at low levels at T2, and one DEG encoding EIN3 was upregulated at T3 despite the significant decrease in ambient temperature. These results imply that ETH might mediate tuber development during the final stage of tuberization.

Transcription factors are well known to be involved in plant growth and tuber formation, including starch biosynthesis, regulation of fruit size, formation of cambium meristems, and hormone biosynthesis [45,51,52]. Several MADS-box genes have been shown to be highly expressed in tubers, including *MADS3*, *MADS4*, *AGL24*, *MDAS11*, *MADS16*, *MADS1*, *SDR1*, *SOC1*, and *FUL1* [53,54,55,56]. In turn, Ku et al. [53] observed that IbMADS1-transformed potatoes exhibited tuber morphogenesis in fibrous roots, and Noh et al. [55] reported that *SRD1* plays a role in the formation of storage roots by activating the proliferation of cambium and metaxylem cells to induce initial thickening growth of storage roots. In our study, 22 MADS-box DEGs were identified and 13 of them were upregulated during ‘Tuershao’ tuberization. These results indicate that MADS-box TFs might act as positive regulators in storage root formation.

Several TFs have been found to participate in starch biosynthesis. Thus, for example, in maize, MYB14 and NAC126 function as key regulators of ZmBT1 and are closely related to starch biosynthesis [57,58]. In turn, overexpression of maize NAC36 upregulates the expression of many starch synthesis-related genes in the endosperm [59]. In our study, the expression of TF genes exhibited different expression patterns. Among these TFs, WRKY DEGs were strongly downregulated and more than half of MYB and NAC DEGs were downregulated during ‘Tuershao’ tuberization. These results imply that WRKY TFs might be negative regulators of tuber formation. However, further investigation is needed to explore the potential roles of these TFs in the development of storage roots.

## 4. Materials and Methods

### 4.1. Plant Material, Sampling, and Soil Physical Properties

Plants of the ‘Tuershao’ cultivar were cultivated in Ya’an city, Sichuan Province (30°06′ N, 102°75′ E). According to the experience of local farmers, tuberization of ‘Tuershao’ plants started from October to December. Thus, the tuber materials were collected on September 14th (T1), November 9th (T2), and December 24th (T3). All samples were frozen in liquid nitrogen and stored at −80 °C for metabolome (six replicates) and RNA-seq (three replicates) analysis at Novogene Science and Technology Co., Ltd. (Beijing, China. http://www.novogene.cn/, accessed on 25 May 2022). The W.E.T sensor kit was used to detect water content (Wet), electrical conductivity (EC), and temperature (T) at three tuber-growth stages (T1, T2, and T3).

### 4.2. Microscopy of Tuber and Root

Samples at the T2 and T1 stages were selected to study detailed anatomical structures at the cellular level. An improved sectioning method was applied, as described by [60]. Nikon Eclipse E100 (Tokyo, Japan) and Nikon DS-U3 (Tokyo, Japan) were used to scan the anatomical structures.

### 4.3. Starch Content Detection

The starch content of ‘Tuershao’ tuber in three stages was determined using the colorimetric method reported by previous study [15]. The starch content could be considered as the sum of amylose and amylopectin.

### 4.4. RNA-Seq Analysis

#### 4.4.1. RNA Quantification and Library Preparation

RNA integrity was assessed using the RNA Nano 6000 Assay Kit of the Bioanalyzer 2100 system (Agilent Technologies, Santa Clara, CA, USA). Total RNA was used as the input material for RNA sample preparation. Briefly, mRNA was purified from total RNA using poly T oligo-attached magnetic beads. Fragmentation was performed using divalent cations at high temperature in the First Strand Synthesis Reaction Buffer (5×).

#### 4.4.2. Clustering and Sequencing (Novogene Experimental Department)

Clustering of the index-coded samples was performed on a cBot Cluster Generation System using TruSeq PE Cluster Kit v3-cBot-HS (Illumina, San Diego, CA, USA). After cluster generation, the library preparations were sequenced on an Illumina Novaseq platform, and 150 bp paired-end reads were generated.

#### 4.4.3. Quality Control and Transcriptome Assembly

Raw data (raw reads) of fastq format were first processed using in-house Perl scripts. Additionally, Q20, Q30, and GC contents of the clean data were calculated. Transcriptome assembly was accomplished using Trinity [61] with min_kmer_cov set to 2 by default and all other parameters set to the default values.

#### 4.4.4. Gene Functional Annotation and Differential Gene-Expression Analysis

Then, clean reads were mapped to the reference genome which was published in the previous study [16].

Differential expression analysis of two conditions/groups (two biological replicates per condition) was performed using the DESeq2 R package (1.20.0). Genes with an adjusted *p*-value< 0.05, found by DESeq2, were assigned as differentially expressed genes (DEGs).

### 4.5. Metabolome Analysis

#### 4.5.1. Metabolite Extraction

Tissue samples (100 mg) were individually ground under liquid nitrogen and homogenates were resuspended by vortexing with 500 μL of prechilled 80% methanol and 0.1% formic acid. After centrifuged at 15,000× *g*, at 4 °C for 20 min, the supernatants were injected into the LC-MS/MS system. The blank sample was a 53% methanol aqueous solution containing 0.1% formic acid instead of the experimental sample.

#### 4.5.2. Metabolite Identification and Quantification

The detection of the experimental samples using multiple reaction monitoring (MRM) was based on a novogene in-house database. The data files generated by HPLC-MS/MS were processed using the SCIEX OS Version 1.4 to integrate and correct the peaks. The main parameters were set as follows: minimum peak height, 500; signal/noise ratio, 10; and Gaussian smooth width, 3. The area of each peak was considered as representative of the relative content of the corresponding substances.

#### 4.5.3. Data Analysis

The metabolites identified were annotated using the KEGG database (http://www.genome.jp/kegg/, accessed on 25 May 2022), the HMDB database (http://www.hmdb.ca/, accessed on 25 May 2022), and the Lipidmaps database (http://www.lipidmaps.org/, accessed on 25 May 2022). Principal component analysis (PCA) and partial least squares discriminant analysis (PLS-DA) was performed using metaX (a flexible and comprehensive software for processing metabolomics data). |Log2 (fold change)| ≥ 1, *p*-value < 0.05, and variable importance in project (VIP) ≥ 1 as thresholds for differentially accumulated metabolites (DAMs).

### 4.6. K-Mean Cluster Analysis

The tendency of DAMs was determined by K-means cluster analysis using R software. Then, DAMs in each group were classified into different classes, and a heat map was created to show the number of DAMs in each class.

### 4.7. qRT-PCR Validation

For PCR validation, thirteen genes were selected, and primers were designed using the primer 3 Online Web site (https://primer3.ut.ee/, accessed on 25 May 2022). All primer sequences used in our study are listed in Table 1. RNA was extracted using the Plant RNA Kit (OMEGA, Norcross, GA, USA). Then, the Prime Script™ RT reagent Kit with gDNA Eraser (TaKaRa, Tokyo, Japan) was used to remove genomic DNA contamination and transform RNA into cDNA. The qPCR was conducted using SYBR Premix Ex TaqII (Tli RNaseH, TaKaRa) on a CFX96 Real-Time System C1000 Thermal Cycler (Bio-Rad, Hercules, CA, USA). Three replicates were performed for three separate RNA extracts from three samples, and the results were calculated using 2^−ΔΔCT^.

### 4.8. Cis-Element Analysis and Conserved Motif Analysis of DEGs

CDS sequences were submitted to MEME online tool (https://meme-suite.org/, accessed on 25 May 2022) to analyze conserved motifs. The number of motifs was set to 10, the minimum width of motifs was set to 6, and the maximum was set to 50 [62]. The upstream sequences (2000 bp) of the start codon of DEGs were extracted using TBtools software [63], then the sequences were submitted to PlantCare online website (http://bioinformatics.psb.ugent.be/webtools/plantcare/html/, accessed on 25 May 2022) to query the cis-acting elements. The results were downloaded and TBtools software was used to visualize the final image [63].

## 5. Conclusions

The high tuber-yield chayote cultivar, ‘Tuershao’ may be developed as a new tuber crop. Transcriptome and metabolome analyses were conducted using ‘Tuershao’ tubers collected at three different growth stages to characterize the patterns of starch biosynthesis, hormone biosynthesis, signal transduction, cell division, tuberization signal complex, and transcription factor regulators. ‘Tuershao’ plants produce edible tubers once a year from October to December in the Sichuan province. To harvest ‘Tuershao’ edible tubers twice a year, the identification of genes and environmental signals that promote tuber formation is an important and challenging goal. Our findings provide an overall gene expression and metabolite transformation network of tuberization of *S. edule* roots. However, additional studies are needed to elucidate the function of these candidate genes. Future research should focus on the genes and environmental signals that control tuber formation in the roots of chayote.

## Figures and Tables

**Table 1:** Primer sequence.

Gene ID	Sequence	Function Describe	Gene ID	Sequence	Function Describe
*Sed0004735*	GGCGTATCATGTCCTCGATT	14-3-3	*Sed0005727*	TTAGGTTGCTTTCTAGCAGGAG	SUT
	GGGATGAGGTGCTTGTCAAT			AGTTGAAGGTGTAGGAAACAGC	
*Sed0023815*	AGATTGCGGATTTGTTGGAC	KNOX	*Sed0018997*	AAGACGAAGAGCGTAGAGTTCA	SWEET
	AGGCCTTTTGAGATCCGATT			GCGATCTCGGTAGATGAAGTAG	
*Sed0014035*	CAAGCTGGAGAAGGATCTGC	MADS-box	*Sed0018484*	AGCTCTACAAAGGAAGGGAAAC	SSY
	CAAAAGGTGACTTGGCGATT			CTCCACTTCAATCTCTCGTAGC	
*Sed0000607*	TGCTGAACTCTGCTGTTGCT	WRKY	*Sed0027566*	TGTAGAAACGATGGAGAGAGGT	SSY
	GGCTGAGGTTGTTCATGGTT			GAAGACGACTCGAGCTTTAGAA	
*Sed0017893*	GGTGAAGAACAGCTGCAACA	AUX1	*Sed0019952*	CTAGGCCAGGACAATATGAAAG	SUS
	CTTCCCATCAGCCCATAGAA			CCTATCTGCAAGGAAACCTATG	
*Sed0001824*	CTCAATCCTAAAGCCAACAGAG	Actin	*Sed0007002*	AAGACGCCTTTCTACACATCTC	glgC
	AGTGTGACTAACACCATCACCA			TCAGTTTGGTAACTGTCTGCAC	
*Sed0004014*	GACCTTCCTCTTGAGCCTATCT	SWEET	*Sed0010193*	GTCCTACAACAACCACTCAGGT	BEL1
	ATTGTACTATCCCCAACACAGC			ATGCTGTTGTAACCCTAACGTC	
*Sed0007063*	GAGGATATACTTGCGCTCAGTC	SUT	*Sed0003801*	AGTGAGAAGCGCCTATATCTTG	CDCP
	ACCAATGCCTGTTACTATGACC			GTGCGACGGTCTATGAGTAGAT	

## Data Availability

Transcriptome sequencing data are available from the NCBI under project ID PRJNA842936.
